# Wnt Signaling in Cancer Metabolism and Immunity

**DOI:** 10.3390/cancers11070904

**Published:** 2019-06-28

**Authors:** Sara El-Sahli, Ying Xie, Lisheng Wang, Sheng Liu

**Affiliations:** 1 Department of Biochemistry, Microbiology and Immunology, Faculty of Medicine, University of Ottawa, 451 Smyth Road, Ottawa, ON K1H 8M5, Canada; 2 Ottawa Institute of Systems Biology, University of Ottawa, 451 Smyth Road, Ottawa, ON K1H 8M5, Canada; 3 China-Canada Centre of Research for Digestive Diseases, University of Ottawa, 451 Smyth Road, Ottawa, ON K1H 8M5, Canada; 4 Institute of Digestive Diseases, Longhua Hospital, Shanghai University of Traditional Chinese Medicine, 725 South Wanping Road, Shanghai 200032, China; 5 Institute of Chinese Traditional Surgery, Longhua Hospital, Shanghai University of Traditional Chinese Medicine, 725 South Wanping Road, Shanghai 200032, China; 6 Ottawa Hospital Research Institute, Faculty of Medicine, University of Ottawa, Ottawa, ON K1H 8M5, Canada; 7 Regenerative Medicine Program, Ottawa Hospital Research Institute, Ottawa, ON K1H 8L6, Canada

**Keywords:** Wnt, cancer, glycolysis, glutaminolysis, lipogenesis, metabolic negative feedback, dendritic cells, T cells, cancer immunotherapy

## Abstract

The Wingless (Wnt)/β-catenin pathway has long been associated with tumorigenesis, tumor plasticity, and tumor-initiating cells called cancer stem cells (CSCs). Wnt signaling has recently been implicated in the metabolic reprogramming of cancer cells. Aberrant Wnt signaling is considered to be a driver of metabolic alterations of glycolysis, glutaminolysis, and lipogenesis, processes essential to the survival of bulk and CSC populations. Over the past decade, the Wnt pathway has also been shown to regulate the tumor microenvironment (TME) and anti-cancer immunity. Wnt ligands released by tumor cells in the TME facilitate the immune evasion of cancer cells and hamper immunotherapy. In this review, we illustrate the role of the canonical Wnt/β-catenin pathway in cancer metabolism and immunity to explore the potential therapeutic approach of targeting Wnt signaling from a metabolic and immunological perspective.

## 1. Introduction

The highly conserved Wingless (Wnt) signaling pathway is important in embryonic development, stem cell maintenance, and wound healing [1]. Wnt signaling pathways have been characterized into the canonical or β-catenin dependent pathway, and the non-canonical or β-catenin independent pathway. Although these two pathways converge, the non-canonical is relatively less characterized, and more focus has been placed on the canonical pathway, where the primary effector is the protein, β-catenin. Wnt proteins regulate different cellular processes including, but not limited to, cell proliferation, cell fate determination, motility, and stem cell renewal. This pathway is predominant and significant and requires tight regulation as any change could cause pleiotropic human disorders [1,2]. Indeed, aberrant Wnt signaling has been implicated in tumorigenesis and cancer progression in many cancers, such as breast cancer [3,4], colorectal cancer [5], thyroid cancer and others [6]. 

Wnt ligands are cysteine-rich secreted glycoproteins that are released into the extracellular milieu and act in a paracrine and an autocrine manner. Wnt binding to the Frizzled (Fzd) receptor and low-density lipoprotein receptor-related protein (LRP) co-receptors triggers a series of events that inhibit the β-catenin destruction complex. The destruction complex is composed of adenomatosis polyposis coli (APC), glycogen synthase kinase 3 (GSK3) and Axin2, and is inactivated through the recruitment of a modular protein called Disheveled (Dvl). The destruction complex is in its active form phosphorylates β-catenin and tags it for ubiquitination and degradation by E3 ligase. Therefore, Wnt binding and subsequent pathway activation allows β-catenin to accumulate in the cytoplasm and translocate to the nucleus where it interacts with T cell factor/lymphoid enhancer factors (TCF/LEF, transcription factors), coactivators CREB-binding protein (CBP) and P300. As a result, it induces the transcription of extensive target genes, and is involved in a wide range of biological processes [1,7,8,9].

Although Wnt activation is highlighted in many cancers [7], it has also been found to suppress other cancers [10,11,12]. It has been well established that Wnt activation induces the expression of stemness genes *c-Myc (a regulator gene and proto-oncogene)*, *Nanog (a transcription factor sustaining pluripotency of embryonic stem cells)*, *Oct4 (octamer-binding transcription factor 4)*, *Sox2 (sex determining region Y-box 2)*, and cancer stem cell-associated genes *CD44 (cluster of differentiation 44)*, *Snai1 (a zinc finger protein regulating epithelial to mesenchymal transition)*, *Twist (a basic helix-loop-helix transcription factor)*. Wnt pathway is upregulated in most patients with breast cancer and has been associated with poor survival [13,14]. Therefore, the Wnt pathway is considered as an attractive therapeutic target to reduce tumor progression and growth [7,15]. Targeted therapies directly or indirectly against different Wnt pathway components have shown pre-clinical success and are currently being tested in the clinic trials (Table 1). 

In recent years, the Wnt pathway has been linked to cancer metabolism and cancer immunotherapy. Although the role of Wnt/β-catenin in liver metabolism [31] and intestinal homeostasis [32] is well established, its role in the reprogramming of cancer cell metabolism has been the subject of inquiry over the last decade. Here, we outline cancer cell reliance on metabolic alterations, highlighting the significance of the Wnt pathway in cancer metabolism. Furthermore, we summarize recent studies regarding Wnt signaling and cancer immunotherapy and their potential applications. 

## 2. Wnt Signaling in Cancer Glycolysis 

**Figure 1:**
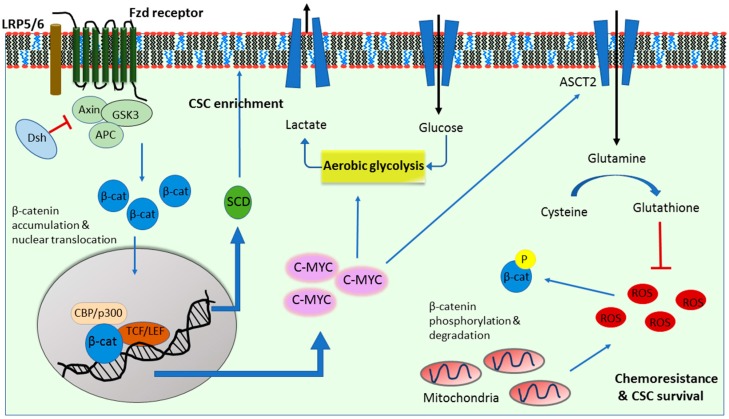
Overview of the role of the canonical Wnt/β-catenin pathway in cancer metabolism. Activation of Wnt signaling requires the binding of Wnt glycoproteins to the frizzled (Fzd) receptor and the low-density lipoprotein receptor-related protein (LRP5/6) co-receptor. Receptor activation leads to the inhibition of the destruction complex, Axin2/APC/GSK3 through the recruitment of disheveled (Dvl). β-catenin can then accumulate in cytoplasm and translocate to the nucleus where the TCF/LEF family of transcriptional factors activate a wide range of Wnt target genes. Through c-MYC, Wnt controls the increased aerobic glycolysis, glutamine transporter ASCT2 levels and subsequent glutathione (GSH) production, which is implicated in cancer chemoresistance and cancer stem cell (CSC) survival via the inhibition of reactive oxygen species (ROS). Wnt also upregulates stearoyl-CoA desaturase-1 (SCD), in particular SCD1, which has been considered as the hallmark for CSC enrichment.

Normal cellular metabolism involves the conversion of glucose to pyruvate for minimal ATP production in a process known as glycolysis. Pyruvate is subsequently converted into acetyl CoA which undergoes a series of reactions in the citric acid cycle (TCA) of the mitochondria. Throughout the TCA process, NADH (nicotinamide-adenine dinucleotide, reduced) and FADH2 (a flavin adenine dinucleotide) are produced to undergo oxidative phosphorylation in the electron transport chain (ETC), generating the majority of the ATP. Pioneering research into the redirected metabolism of cancer cells was carried out by Warburg in the early 1900s. He observed that cancer cells underwent glycolysis instead of oxidative phosphorylation to sustain their energy demand even in the presence of adequate supplies of oxygen, in the phenomenon known as aerobic glycolysis or the “Warburg effect” [33]. The reliance on glucose metabolism and low dependence on mitochondrial activity was deemed characteristic of cancer cells. It is thought that mitochondrial dysfunction in cancer cells is one of the many reasons that drives the cancer cell preferences to a more glycolytic phenotype, favoring an increased uptake of glucose and conversion of pyruvate into lactate to generate energy [34]. Lactate production results in the acidification of the microenvironment which further helps tumor migration and invasion. It is also well established that a glycolytic switch in cancers and increased glucose uptake correlates with a poorer prognosis and more aggressive phenotypes [35]. As such, more research has investigated the distinct mechanisms and key players responsible for this metabolic switch in cancer cells, implicating Wnt/β-catenin signaling in these changes (Figure 1). 

**Figure 2:**
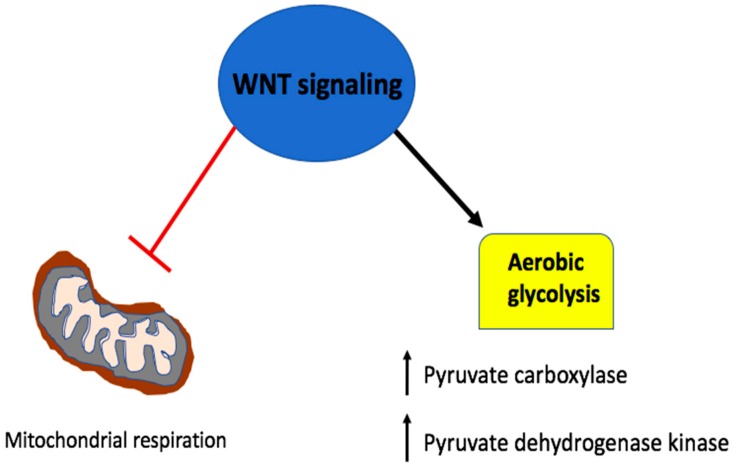
Wnt signaling in the metabolic reprogramming of cancer cells. The Wnt pathway upregulates aerobic glycolysis in part through its control of pyruvate carboxylase and pyruvate dehydrogenase kinase, enzymes that shift the metabolic requirement away from the mitochondrial oxidative phosphorylation (OXPHOS).

A study by Lee et al. in 2012 showed that Wnt/β-catenin signaling induced an increase in glucose uptake and suppressed mitochondrial respiration [36]. They further revealed a Wnt-induced upregulation of pyruvate carboxylase, an enzyme that converts pyruvate to oxaloacetate to support cell proliferation [36]. Pate et al. then showed that Wnt pathway disruption led to decreased reliance on aerobic glycolysis by cancer cells [37]. They suggested that this effect was at least in part due to Wnt controlled pyruvate dehydrogenase kinase (PDK1), an enzyme that inhibits mitochondrial oxidative phosphorylation (OXPHOS) by reducing the conversion of pyruvate into acetyl-coA and thereby maintaining the glycolysis dependent nature of tumor cells (Figure 2) [37]. In triple negative breast cancer (TNBC, an aggressive subtype of breast cancer), the Wnt ligand, Wnt5B was shown to suppress mitochondrial function through the Wnt/β-catenin target gene *c-MYC* [38]. Immunohistochemistry of 142 breast tumor tissue samples revealed a positive correlation between MYC and mitochondrial regulator MCL1 [38,39]. C-MYC, known as a regulator of aerobic glycolysis, in turn acts as a transcription factor to mediate Wnt/β-catenin in the control of cancer cell metabolism (Figure 1) [37,40]. 

Aerobic glycolysis also contributes to the maintenance of stemness in cancer stem cells (CSCs), which has been linked to Wnt signaling. The production of reactive oxygen species (ROS) as a result of mitochondrial respiration impairs the self-renewal ability of stem cells, and explains the shift to aerobic glycolysis undertaken by CSCs. Indeed, poorly differentiated cancer cells rely more on increased glucose uptake than their differentiated counterparts. A study by Peng et al. showed that PDK1, which was regulated by Wnt signaling [37], was crucial in maintaining CSC populations in breast cancer [41]. While Wnt/β-catenin signaling is crucial in CSC self-renewal, it remains poorly understood whether Wnt-mediated regulation of cancer metabolism plays a key role in maintaining CSC population. However, a recent study by Deshmukh et al. suggested that the Wnt antagonist, secreted frizzled-related protein 4 (sFRP4) regulates CSC metabolism, where glucose-mediated increase in CSC viability was diminished by sFRP4 treatment [42]. Whether this CSC metabolism was controlled solely through Wnt signaling and whether there are other players involved in downstream or upstream of the Wnt pathway remains elusive. As c-MYC is also a key regulator of CSCs [43], the exact role of Wnt upstream/downstream *c-MYC* gene expression in CSC metabolism remains to be explored. 

## 3. Wnt Signaling in Cancer Glutaminolysis 

In addition to a shift to aerobic glycolysis, cancer cells rely on the provision bulk quantities of amino acids as essential precursors required for cell survival and growth, and to replete the TCA cycle [44]. Indeed, increased glutamine uptake is well documented in many different cancers including breast cancer [45,46]. However, this seems to be cancer-subtype dependent. For example, triple-negative breast tumors are more heavily dependent on glutamine and therapeutic approaches comprising glutamine-targeting therapies may be most effective [47]. Glutamine plays significant roles in cell proliferation, survival and migration [48,49]. In particular, glutamine undergoes glutaminolysis, a process by which it is converted to glutamate and α-ketoglutarate to replenish the TCA cycle, support protein synthesis and produce glutathione (GSH). α-ketoglutarate could also supplement aerobic glycolysis as it can be converted to malate and then to pyruvate [50]. As such, glutaminolysis is considered a metabolic adaptation strategy undertaken by cancer cells to supply nitrogen to sustain their rapid division and energy requirement [51]. 

It has long been established that the Wnt/β-catenin pathway plays an important role in glutamine metabolism. Cadoret et al. showed that β-catenin activated genes were involved in glutamine uptake and metabolism [52]. While the exact mechanism by which Wnt/β-catenin influences glutaminolysis is still incompletely understood, c-MYC seems to play a crucial role [53]. C-MYC has been shown to induce the expression of genes involved in glutamine metabolism, such as the glutamine transporter ASCT2 (or SLC1A1) and glutaminase [48,49,53]. Furthermore, Wu et al. identified β-catenin in the MYC–mediated control of glutamine metabolism and glutaminolysis [54]. Although NF-κB (nuclear factor kappa-light-chain-enhancer of activated B cells) was suggested as a regulator of glutaminolysis in transformed fibroblasts and breast cancer cells [49], the role of Wnt signaling in this regulation remains unexplored. 

Glutathione (GSH), a product of glutamine metabolism, plays a role in the chemoresistance of cancer cells [55] and CSCs [56], which has also been associated with Wnt signaling [57]. The role of GSH seems to be especially important in CSC chemoresistance, as it was shown that CD44+, a CSC surface marker, associates with glutamine-cysteine transporter and promotes the synthesis of GSH [56]. GSH, in turn, helps protect the cells against oxidative stress by neutralizing ROS as well as activating cell survival pathways [58]. This served as preliminary evidence, suggesting that GSH plays a role in the maintenance of the CSC population in a tumor. Recently, an interesting study by Miran et al. found that the depletion of GSH resensitized the CSC population to therapy in breast cancer [59]. Pre-treatment with a GSH inhibitor led to decreased tumor growth in an in vivo mouse model [59]. Indeed, preclinical investigation of drugs targeting glutaminolysis and glutamine metabolism have garnered success as metabolic therapies in breast cancer, and some have moved to clinical trials [60]. A study by Liao et al. found that glutamine deprivation in CSCs resulted in decreased GSH and increased β-catenin phosphorylation and sequestration in addition to decreased Wnt signaling activity. Glutamine’s regulation of stem-like cancer cells was shown to partially occur through ROS-mediated β-catenin phosphorylation and degradation [57]. This suggests that targeting GSH production could also inhibit the CSC population by downregulating Wnt/β-catenin activity. Interestingly, c-MYC levels were unaffected upon glutamine withdrawal in these cancer stem cells, indicating no feedback loop in c-MYC regulation of glutamine metabolism [57]. The above studies together suggest that Wnt signaling and GSH production regulate each other and their co-inhibition would be the most effective as it would alter glutamine’s regulation of CSCs and reduce GSH production, thereby sensitizing the cells to chemotherapeutic agents.

## 4. Wnt Signaling in Cancer Lipogenesis 

Wnt signaling has also been associated with cancer lipogenesis. Glutamine could alternatively be pushed towards reductive carboxylation, where it is converted to α-ketoglutarate for the provision of acetyl-CoA for de novo lipogenesis. De novo lipogenesis supplies the growing cells with high energy levels as well as building blocks for essential cellular components [61]. Abnormal levels of lipids in cells correlate with cancer progression in a multitude of different tumors. For example, in breast cancer, there is a higher accumulation of cholesterol esters, free fatty acids, and phospholipids, which have been shown to promote tumorigenicity and cancer cell invasion [62,63]. Cholesterol ester-rich tumors were associated with increased breast tumor proliferation and necrosis [62]. Furthermore, linoleic acid was shown to induce breast cancer cell migration through plasminogen activator inhibitor-1 (PAI-1) and SMAD4 [63]. In a recent study, Yao et al. found that canonical Wnt signaling through MYC promotes the conversion of triacylglycerol to phospholipid and increases unsaturated fatty acyl groups in phospholipids [64]. This lipid metabolism remodeling is extensively used by cancer cells as unsaturated fatty acids are crucial for the cell membrane maintenance, energy storage and signaling [65]. Additionally, unsaturated fatty acids are linked to the stem-like characteristics in ovarian cancer [66]. β-catenin knockdown in breast cancer cells was also shown to result in a reduction in key lipogenic enzymes such as citrate carrier, acetyl-CoA carboxylase and fatty acid synthase [67], which emphasizes the role of Wnt/β-catenin signaling in de novo lipid synthesis. 

Increasing evidence has highlighted the role of lipid metabolism in CSC survival and maintenance. There are subtle differences in the lipid metabolism alterations between CSC and non-CSC populations. In breast cancer, the fatty acid β-oxidation enzyme, carnitine palmitoyltransferase I (CPT1), is shown to be elevated in the CSC population more than the non-CSC population [68]. In these CSCs, the key player between Wnt signaling and lipid metabolism could be stearoyl-CoA desaturases (SCDs). SCDs catalyze lipid desaturation and growing evidence identifies SCDs as a hallmark of CSCs [69,70,71]. The connection between the Wnt pathway and SCDs has been well established, where β-catenin was found to increase the sterol regulatory element binding protein 1 (SREBP-1)-mediated expression of the major forms of SCD (i.e., SCD1 and SCD2) in CSCs. SCD silencing upregulated the expression levels of β-catenin, which was overcome after the addition of the product of the SCDs, monounsaturated fatty acids (MUFAs) [71]. In a different study, MUFAs were deemed to be crucial in Wnt ligand production and secretion [72]. Lastly, SCD1 was shown to regulate the Hippo/YAP pathway, a key CSC-associated oncogenic pathway, at least, in part, through Wnt signaling [73]. Dual inhibition of Wnt and YAP has been shown to delay the growth of triple-negative breast cancer in both mesenchymal and epithelial states [74]. The above studies together suggest that both Wnt and YAP pathways could contribute to lipid metabolic reprogramming in cancer cells, and inhibition of both could be more effective for targeting CSCs. 

## 5. Wnt Signaling in A Metabolic Negative Feedback Loop

The existence of a negative feedback loop adds a layer of complexity to the role of Wnt/β-catenin in the regulation of metabolism. The electron transport chain enzyme, succinate dehydrogenase suppresses Wnt induced tumor progression by activating glycogen synthase kinase-3β (GSK3B), promoting the destruction complex formation and thereby preventing β-catenin driven transcription [75]. The key gluconeogenesis enzyme, Fructose-1,6-bisphosphatase (FBP1), was found to decrease glucose uptake and lactate production as well as increase mitochondrial OXPHOS in a β-catenin dependent manner in breast cancer cells [76]. Similarly, a nuclear protein called Chibby was discovered as a β-catenin-associated antagonist that inhibits β-catenin-mediated transcriptional activation by competing with its binding to LEF-1 in the nucleus [77]. Chibby was shown to suppress aerobic glycolysis by downregulating the Wnt induced PDK1 upregulation [78]. Inducing these natural metabolic Wnt antagonists could be a potential therapeutic avenue to counteract metabolic reprograming in cancer cells. 

The effect of mitochondrial OXPHOS on cancer cells and Wnt signaling has been highly debated. A recent study has shown that mitochondrial function promotes tumorigenesis in colon cancer through hypoxia-inducible factors-1 (HIF-1) and Wnt signaling. Inhibition of mitochondrial function led to an increase in the level of TCA intermediate, α-ketoglutarate, which was subsequently identified as a key player that suppresses Wnt signaling [79]. ROS formation after FBP1 knockdown was shown to interfere with nuclear activity of β-catenin, reducing tumorigenesis and the cancer stem cell population in TNBC [79]. However, the opposite effect was seen with luminal subtypes of breast cancer where FBP1 knockdown increased CSC enrichment [76]. Similarly, in lung cancer, ROS production as a result of glutamine deprivation seems to increase β-catenin phosphorylation and thus, downregulates its transcriptional activity [57]. Therefore, the effect of mitochondrial OXPHOS on Wnt/β-catenin signaling is likely tumor and subtype dependent, warranting further studies. 

It has recently been shown that the essential amino acid methionine plays a crucial role in regulating the Wnt endolysosomal activity through 1 carbon (1-C) metabolism, a metabolic process that transfers methyl groups to various substrates. Inhibition of 1-C metabolism is suggested to halt Wnt-driven cancers. The chemotherapeutic agent, methotrexate, inhibits protein arginine methyl transferase 1 (PRMT1) and Wnt-induced endolysosomal activity. PRMT1 is crucial in stem cell activity and highly expressed in the Wnt dependent stem cells of the intestines [80], suggesting a potential role of Wnt signaling in PRMT1 and CSC metabolism. 

## 6. Wnt Signaling in Cancer Immunotherapy 

The tumor microenvironment (TME) is composed of tumor cells, fibroblasts, stromal cells, vasculature, immune cells and extracellular matrix [81]. The infiltration of immune cells plays multiple roles in either the promotion or delay of tumor progression. Anticancer immunity is largely dependent on CD8+ cytotoxic T lymphocytes, which were first shown to recognize tumor antigens in melanoma [82]. Active CD8+ T cells produce cytokines, cytotoxic perforins and granzymes, which target tumor cells mainly by promoting cell apoptosis. As such, tumors with a higher T-cell infiltration typically have a favorable prognosis and better response to treatment [83,84]. Dendritic cells (DCs) are the primary antigen presenting cells (APCs) of the immune system. DCs uptake and present tumor antigens after which, they activate CD8+ T cells and/or CD4+ T cells. DCs present tumor antigens to CD8+ T cells to elicit their cytotoxic effect [85]. On the other hand, DCs in a regulatory state could stimulate regulatory CD4+ T-cells known as Tregs (T regulatory cells), which mediate immune suppression in tumors [86]. Cancer cells create an inhibitory TME to evade immune surveillance by downregulating tumor antigenicity, suppressing cytokines and factors, and increasing the Treg population.

Recent research attempts to find new therapeutic approaches to eliciting anti-tumor immune responses due to the limited success for considerable cancers in the clinic. Wnt/ β-catenin signaling is implicated in cancer immunotherapy as well. Wnt activation influences anti-tumor immunity. Indeed, a plethora of evidence has implicated Wnt/β-catenin signaling, as it seems to play an important role in cancer immunotherapy. Aberrant Wnt signaling is well documented in immune evasion, and tumors with high Wnt signaling displayed lower immune cell infiltration [87]. 

**Figure 3:**
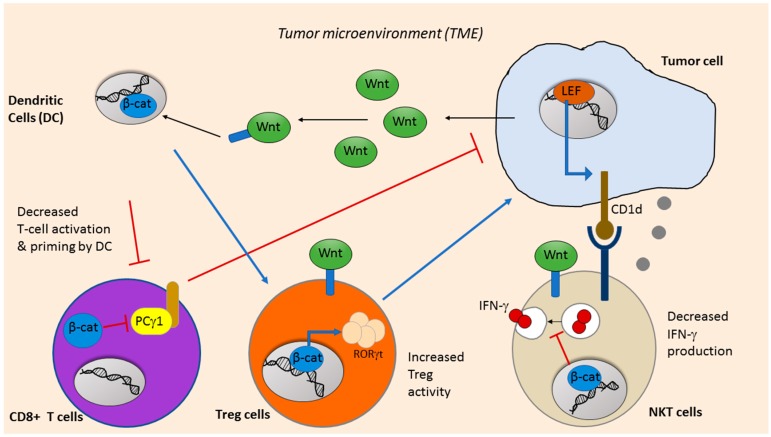
General schematic of Wnt signaling in tumor induced immunosuppression. Wnt ligands released by the tumor cells in the microenvironment influence dendritic cell (DC) function and chemokine release, which, in turn, suppresses priming and activation of CD8+ cytotoxic T-cells and prevents their anti-cancer action. β-catenin in CD8+ T cells decreases phospholipase C-γ1 (PCγ1) activity which is essential to T-cell activation. However, the activation of Wnt/β-catenin in CD4+ regulator T cells (Tregs) could induce the expression of RORγt and promote cancer to evade host immunity. Natural killer T (NKT) cells kill tumor cells indirectly by releasing interferon gamma (IFN-γ) to elicit the response of other immune cells or directly by inducing apoptosis by releasing perforin in a CD1D-dependent manner. While Wnt signaling upregulates CD1D expression, it has also been shown to reduce IFN-γ production.

Immunosuppression in cancer is aided by dysfunctional DC cells in the TME. It has been well established that Wnt signaling regulates DC development and function. Wnt ligands released by tumor cells promote the expression of β-catenin in DC cells to activate Tregs, ablate CD8+ T-cell function and suppress anticancer immunity [88,89]. Hong et al. showed that DCs expressed Wnt co-receptors LRP5 and LRP6, and the specific deletion of LRP5/6 on DC cells delayed tumor growth and enhanced the antitumor immunity [90]. Furthermore, a recent study showed that the Wnt ligand, Wnt1, induced a tolerogenic response in lung cancer. Wnt signaling led to T-cell attenuation by suppressing the CC and CXC motif chemokine transcription in DCs through the downregulation of their transcription factor, Cebpb [91]. Therefore, DCs exposed to high levels of Wnt1 displayed reduced chemokine expression that was crucial for T-cell priming and activation (Figure 3). The administration of RNAi against Wnt was able to increase the cytotoxic T cell population and reduce tumor burden [91]. 

The effect of Wnt signaling on Tregs is controversial with mixed evidence. In the context of inflammation and autoimmune disease, Loosdregt et al. showed that the activation of Wnt signaling reduced Tregs by reducing FOXP3 transcriptional activity [92]. On the other hand, Keerthivasan et al. found that the activation of β-catenin in colon cancer induced the expression of RORγt (Retinoic-acid-receptor-related orphan nuclear receptor gamma) in Treg cells and promoted cancer [93]. Furthermore, in a recent study in colorectal cancer, they identified a set of peptides that inhibit the activity of β-catenin and suppress cancer cell growth. In vivo, these peptides exhibited anti-tumor effects with minimal toxicities by promoting intratumoral infiltration of cytotoxic T cells and reducing Tregs [94]. 

Wnt signaling also plays an important role in T-cell proliferation and differentiation. TCF and LEF exhibit dynamic expression throughout T-cell maturation and constitutive activation of Wnt/β-catenin pathway reduced the expansion of mature cytotoxic T cells [95]. Stabilized β-catenin in T cells inhibited their maturation, differentiation and activation by reducing phospholipase C-γ1 activity and IL-2 production, thus, promoting cancer growth [93,96]. However, increased expression and secretion of Wnt antagonist Dickkopf-related protein 2 (DKK2) by tumor cells inhibited T-cell function and promoted tumor progression in colorectal cancer, independent of β-catenin [97]. This suggests that inhibition of Wnt pathway in different cells could have differential effects on T-cell function and anticancer immunity. 

Natural Killer T (NKT) cells are specialized T cells that share properties of both innate Natural killer (NK) cells and adaptive T cells. Released cytokines from activated NKT cells regulate immune cells (NK cells, T cells and DCs, etc.) in the TME by releasing IFNγ and IL4, causing various anti-tumor responses [98]. In addition, NKT cells recognize glycolipid antigens in a CD1D-dependent manner and can directly kill tumor cells by releasing perforin. Wnt signaling is involved in NKT cell maturity and LEF-1 is known to regulate CD1D gene expression [99]. Recent evidence has also shown that β-catenin is essential in NKT development and differentiation as transgenic β-catenin expression increased the frequency and number of NKT cells that produce more type-2 cytokines [100]. While Wnt/β-catenin signaling is beneficial and required in NKT maturation in some studies, its aberrant activity has also been linked to poor terminal differentiation and function of NKT cells [101]. In addition, Kling et al. found that Wnt ligands and β-catenin activity in NKT cells suppressed IFNγ production [102]. 

Immune evasion in tumors is also driven by their expression of PD-L1 (programmed death-ligand 1) [103], which is recognized by inhibitory immune checkpoint receptor PD-1 on activated T cells. Therapies targeting immune checkpoint inhibitors have garnered recent attention as they were shown to enhance anti-tumor immunity by restoring CD8+ T cell activity and suppressing Tregs. Recent work showed that in specific cell lines, PD-L1 expression correlates with CSC markers [104]. A study by Castagnoli et al. showed that TNBC CSCs upregulated PD-L1. Interestingly, they show that Wnt gene activation was correlated with PD-L1 levels. Through clinical dataset analysis, PD-L1 overexpression was found in TNBC tumors: the more enriched stem cells, the more active the Wnt pathway [103,105]. Additionally, a Wnt inhibitor decreased PD-L1 expression while Wnt agonists were found to enhance PD-L1 at the transcript and protein levels. Lastly, in an *in vivo* model, PD-L1^+^ CSCs were shown to interact with immune cells to regulate immune response [103]. This suggests that targeting Wnt to diminish CSC population could also enhance anti-tumor immune response, however, the exact mechanism by which Wnt upregulates PD-L1 is still unclear. 

## 7. Future Directions: Targeting Wnt Signaling to Inhibit Cancer Metabolism and Enhance Immune Response 

Tumor cell metabolism creates an immunosuppressive environment where increased uptake of nutrients by cancer cells deprives the metabolites required by immune cells in the TME (such as glutamine and glucose) to sustain their survival and expansion. Indeed, recent studies have shown that cellular metabolism also plays a key role in supporting immune cell maintenance and development [106]. Non-specific depletion of nutrients to target metabolic reprogramming of cancer cells could also negatively affect the immune response in the tumor [107,108]. While targeting of cancer metabolism through the Wnt pathway is considered an attractive strategy, the dependence of immune cells on metabolites for their survival and activation could impede clinical application of anti-metabolism drugs. A strategy targeting the metabolic reprogramming in cancer cells as a way to halt tumor growth should take into account the effect on the immune system. Furthermore, different immune cells have different metabolic requirements. For instance, deficiency of the glucose transporter GLUT1 decreases cytotoxic T-cell expansion and function but has little impact on Treg cells, negatively impairing anti-tumor immune response [109]. On the other hand, Tregs rely more on fatty acid oxidation but less on fatty acid synthesis to generate energy [110]. 

An efficient strategy could therefore be to target cancer metabolism while minimizing the effect on immune cells. A possible way to achieve this might be by tackling the acidity of the TME. Targeting increased lactate acid extracellular secretion could potentially boost immune response as the acidity of the TME has been linked to immunosuppression [111,112]. A potential target could be the lactate transporter, monocarboxylate transporter 1 (MCT1) which is upregulated in cancer cells. Since MCT1 was identified as a Wnt pathway target gene [113], the inhibition of Wnt would downregulate MCT1, thereby reducing TME acidity to halt cell migration and metastasis and maintain anti-cancer immunity. Jones et al. recently found that STAT5 is a key note in the activation of CD4+ T cell by regulation of their glycolysis and OXPHOS [114]. It is known that STAT5 activation is downregulated by GSK3β, a component of the destruction complex of β-catenin [115]. As such, inhibition of Wnt effectors β-catenin (downstream of GSK3β) or TCF/LEF, P300, or CBP would target cancer cells while insignificantly or not affecting immune cells in the TME. Further studies of the differential effects of these inhibitors (which are currently in clinical trial for cancer treatment, Table 1) on cancer cells and immune cells may provide new insights and lead to new therapeutic approaches.

PD-L1 was recently shown to be instrumental in metabolic alterations of cancer and immune cells. Checkpoint inhibitors against PD-1 and PD-L1 were shown to diminish glycolysis in cancer cells, thereby restoring glucose in the TME and promoting T-cell function [116]. Inhibition of Wnt signaling was shown to overcome PD-1 inhibitor resistance in breast cancer and reduced tumor growth in vivo. Therefore, the combination of checkpoint inhibitor and Wnt antagonist may increase therapeutic efficacy in the clinic [94].

Amino acid uptake is essential to both tumor progression and immune cell function. While targeting glutamine uptake was shown to decrease cancer cell growth [117,118], glutamine uptake was also crucial for T-cell activation and its inhibition could decrease T cell cytotoxicity [119,120]. However, Wang et al. showed that glutathione released by fibroblasts in the TME contributed to chemoresistance in ovarian cancer, which was overcome by CD8+ T cells after inhibiting glutathione synthesis. This suggests that targeting amino acid metabolism through glutaminolysis could sensitize the cells to chemotherapy and aid in the immune cell response [121]. 

## 8. Conclusions 

Tumor initiation and progression is dependent on many factors, including metabolic reprogramming, immune system evasion and CSC maintenance. While Wnt/β-catenin has been tightly linked with cancer metabolism, much remains unknown about the exact molecular mechanisms underlying. A recurring theme seems to be that Wnt-mediated metabolic control is tumor type dependent. Wnt signaling has also been implicated in immunotherapy. However, due to the complexities of the Wnt pathway and its vast domain of control over immune cell development, inhibiting Wnt could be counterproductive. Designing a therapeutic strategy by inhibition of Wnt signaling to reduce metabolic reprogramming of cancer cell and to increase anti-tumor immunity may need to take into account the tumor type/subtype, the biochemical and mutational analysis of the tumor, the Wnt signaling components to be targeted, and the effect of the Wnt inhibition on cancer cells, CSCs, and the different immune cells. 

## Figures and Tables

**Table 1:** Clinical trials using drugs that are known to reduce Wingless (Wnt) signaling.

Clinical Trial	Cancer Type	Phase	Component Targeted	References
NCT02950259	Breast cancer	I	β-catenin	[16]
NCT02807805	Prostate cancer	II	Dvl & β-catenin	[17,18]
NCT02675946	GI cancer	I	Wnt ligands	[19]
NCT03090165	TNBC	II	β-catenin	[20,21]
NCT02513472	Breast cancer	I	β-catenin	[22]
NCT03355066	Advanced solid tumors	I	Unknown	[23]
NCT01351103	Lung cancer, colorectal cancer, TNBC... etc.	I	Wnt ligands	[24]
NCT02429427	Breast cancer	III	GSK3	[25]
NCT02346032	Biliary Tract Cancer	II	Wnt3 & LRP6	[26]
NCT02005315	Pancreatic Cancer	I	FZD	[27]
NCT01302405	Advanced solid tumors	I	β-catenin/CBP	[28]
NCT02402764	Breast cancer	II	APC	[29]
NCT02426723	Multiple myeloma	I	β-catenin	NCT02426723
NCT02852564	Bladder cancer	I	LEF-1/βcatenin	[30]

Note: Current clinical trials exploring the effects of inhibition of the Wnt/β-catenin pathway in cancers. LRP: Low-density lipoprotein receptor-related protein. DVL2: disheveled. FZD: frizzled receptor. CBP: CREB-binding protein. GSK3: glycogen synthase kinase 3. TNBC: Triple negative breast cancer. GI: gastrointestinal.
